# ‘The phone reminder is important, but will others get to know about my illness?’ Patient perceptions of an mHealth antiretroviral treatment support intervention in the HIVIND trial in South India

**DOI:** 10.1136/bmjopen-2015-007574

**Published:** 2015-11-02

**Authors:** Rashmi Rodrigues, S Poongulali, Kavitha Balaji, Salla Atkins, Per Ashorn, Ayesha De Costa

**Affiliations:** 1 Karolinska Institutet, Stockholm, Sweden; 2 Department of Community Health, St. John's Medical College, Bangalore, Karnataka, India; 3 YRG Care Center, Chennai, Tamil Nadu, India; 4 Department for International Health, University of Tampere School of Medicine, Tampere, Finland

**Keywords:** QUALITATIVE RESEARCH, PUBLIC HEALTH

## Abstract

**Objectives:**

The recent explosion of mHealth applications in the area of HIV care has led to the development of mHealth interventions to support antiretroviral treatment adherence. Several of these interventions have been tested for effectiveness, but few studies have explored patient perspectives of such interventions. Exploring patient perspectives enhances the understanding of how an intervention works or why it does not. We therefore studied perceptions regarding an mHealth adherence intervention within the HIVIND trial in South India.

**Methods:**

The study was conducted at three clinics in South India. The intervention comprised an automated interactive voice response (IVR) call and a pictorial short messaging service (SMS), each delivered weekly. Sixteen purposively selected participants from the intervention arm in the HIVIND trial were interviewed. All participants had completed at least 84 weeks since enrollment in the trial. Perceptions on the usefulness and perceived benefits and risks of receiving the intervention were sought. The interviews were transcribed and analysed using the framework approach to qualitative data analysis.

**Results:**

Despite varying perceptions of the intervention, most participants found it useful. The intervention was perceived as a sign of ‘care’ from the clinic. The IVR call was preferred to the SMS reminder. Two-way communication was preferred to automated calls. Participants also perceived a risk of unintentional disclosure of their HIV status and stigma thereof via the intervention and took initiatives to mitigate this risk. Targeting reminders at those with poor adherence and those in need of social support was suggested.

**Conclusions:**

mHealth adherence interventions go beyond their intended role to provide a sense of care and support to the recipient. Although automated interventions are impersonal, they could be a solution for scale up. Interventions that engage both the recipient and the healthcare provider have greater potential for success. Personalising mHealth interventions could mitigate the risk of stigma and promote their uptake.

**Trial registration number:**

ISRCTN79261738.

**Strengths and limitations of this study**

- This is the first qualitative study in the Indian context designed within a randomised controlled trial that investigates participant perceptions regarding an mHealth adherence support intervention for antiretroviral therapy.
- Qualitative research alongside clinical trials contextualises interventions and facilitates the development of interventions that are effective.
- The framework approach used in the analysis is poised between inductive and deductive approaches and encourages the analysis of the respondents’ perceptions, in light of the researchers’ experiences.
- As the HIVIND trial was ongoing at the time of the interviews, the adherence status of the participants was unknown to the interviewer. Hence, we were unable to relate the experiences of the participants to their adherence within the trial.

## Introduction

The scope of mobile phones in support of healthcare (mHealth) has grown in recent years as evidenced by the expanding body of literature in this field.^1^ ^2^ One of the popular uses of mHealth has been to support the continuum of HIV care and prevention.^3^ ^4^ The technology has been used extensively to spread awareness regarding HIV infection and its prevention,^1^ including the prevention of mother-to-child transmission (PMTCT).^5^ Moreover, mHealth has been found acceptable for communicating laboratory results to HIV-infected individuals, despite sensitivity surrounding the disease.^6^ It is also being studied for HIV counselling and retention^7^ ^8^ in treatment. Supporting adherence to antiretroviral treatment (ART) has been one of the popular uses of the technology, with a number of studies^4^ ^9–12^ showing mixed results.^9^ ^10^ ^13^

Adherence to treatment is a complex^14^ yet essential phenomenon for positive health outcomes, irrespective of the disease. It is key to the success of ART, as optimal adherence defers treatment failure and death in HIV-infected individuals. One of the barriers to adherence is forgetfulness. Several adherence support interventions have been developed to target forgetfulness such as pillboxes, electronic reminders^15^ ^16^ and mobile phone reminders. These interventions are posited within the Behavior Learning Theory (BLT) framework where they are considered as external cues that support medication adherence.^16^

The recently concluded HIVIND randomised controlled trial (RCT) in South India was the first RCT that explored the effect of an mHealth intervention on ART adherence in the Indian context.^17^ The intervention tested in the HIVIND trial comprised an automated interactive voice response (IVR) call and a neutral picture short messaging service (SMS).

Qualitative studies can aid the understanding of the effects of an intervention and the possible pathways through which these effects (or lack of) may be mediated. Such research alongside RCTs can contextualise interventions and facilitate the development of effective interventions.^18^ ^19^ However, qualitative studies alongside RCTs, especially in HIV infection, are few.

We explored participant perceptions of the intervention, including concerns about stigma and intrusion of privacy, and compared the IVR call with the SMS reminder in the Indian context.

## Methods

### Study context

This was a qualitative study within the HIVIND trial. The HIVIND trial was a parallel group open-label trial that studied the effect of mobile phone reminders on adherence to ART and time to first-line ART failure in treatment naïve people living with HIV (PLHIV) in South India. The RCT had three recruitment sites in South India, that is, (1) St. John's Medical College Hospital, Bangalore, a private tertiary-level non-profit healthcare facility, providing care and treatment to approximately 3000 PLHIV/AIDS through a public–private partnership in Karnataka State; (2) KR Hospital, Mysore, a public tertiary-level teaching healthcare facility, providing care and treatment to approximately 10 000 PLHIV in Karnataka State and (3) YRG Care, Chennai, a private healthcare facility, providing care and treatment to approximately 18 000 PLHIV in Tamil Nadu State.

HIV care and treatment in India are provided at no cost or minimal cost to PLHIV through a network of public, private and public–private healthcare facilities. All PLHIV registered within this HIV care network receive counselling support, basic investigations such as CD4 count, treatment and monthly follow-up care. Treatment is initiated at CD4 counts <350 cells/mm^3^.

**Figure 1:**
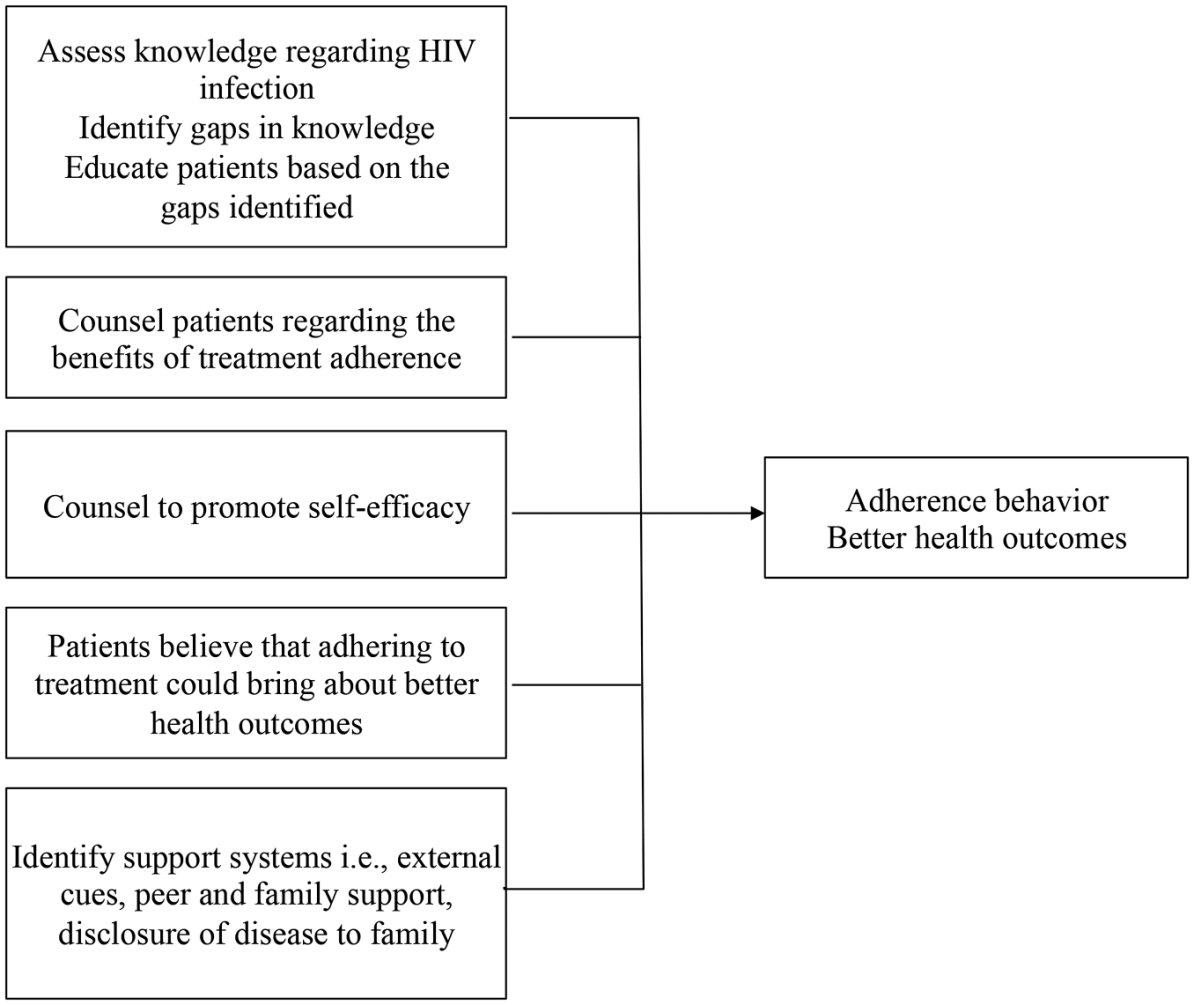
Adherence counselling to support antiretroviral treatment provided to patients in line with Social Cognitive Theory.

Further, PLHIV receive adherence counselling at treatment initiation, and at monthly pill refill visits. The elements of this counselling (figure 1) are in line with the Social Cognitive Theory for behaviour change. According to the existing system, counsellors assess patients’ knowledge regarding their health and educate them based on their knowledge gap. Counselling fosters self-efficacy by engendering the belief that one can independently address adherence-related issues and help identify external systems (antecedents) suitable to support adherence.^20^

*The HIVIND Trial*^19^: The trial tested an mHealth ART adherence support intervention in 631 outpatients aged 18–60 years. Half the participants in the trial (315) received the intervention, while the other half received standard care. All participants, irrespective of receiving the intervention, were followed up quarterly for 2 years or until treatment failure, whichever was earlier. Of the participants in the trial, 502 (79%) were literate, 273 (43%) were female, 398 (63%) were employed and 286 (45%) were from a rural background. The recently reported HIVIND trial results showed no effect of the intervention on adherence.^21^

**Figure 2:**
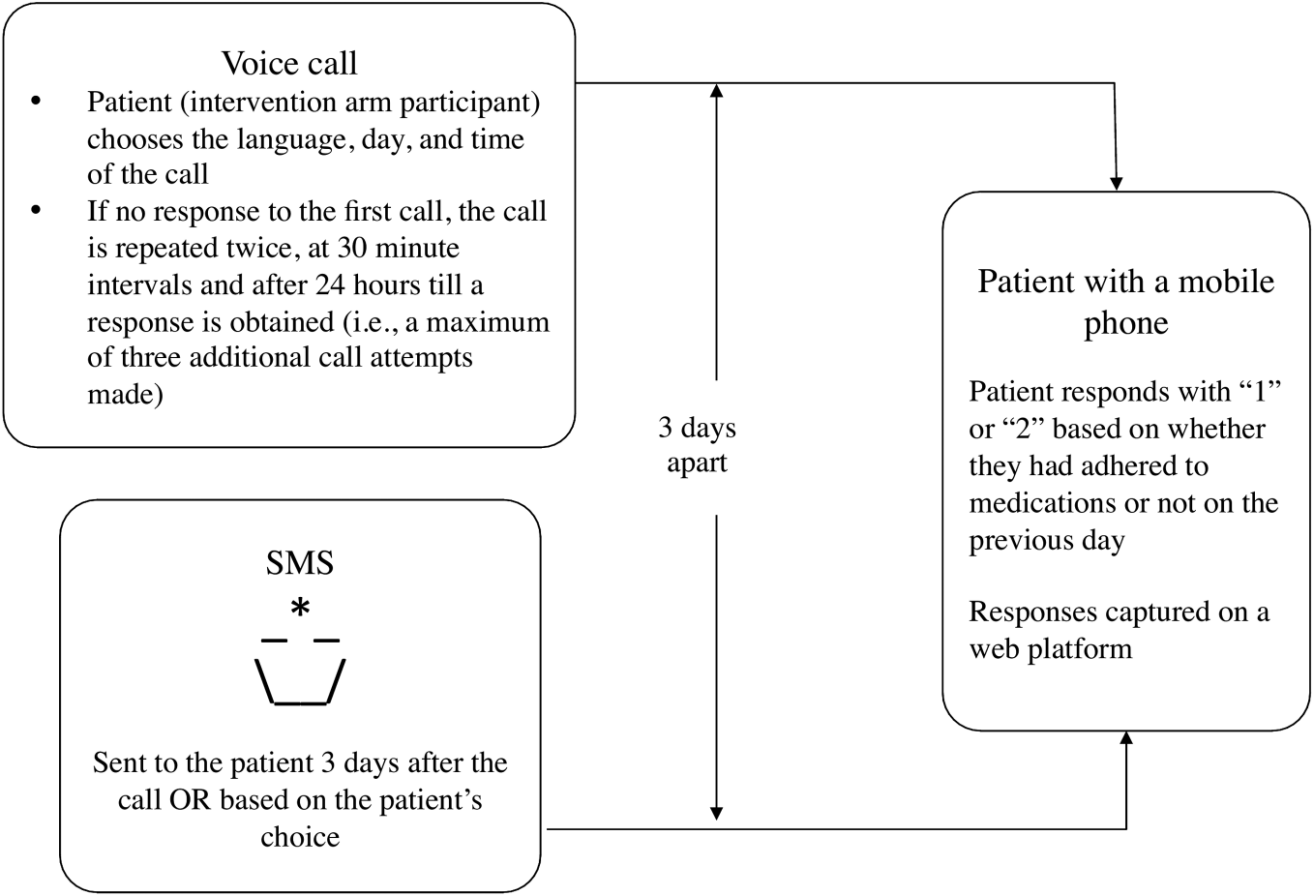
The intervention.

*The mHealth intervention* (figure 2): The trial intervention was designed on the basis of patient preferences and piloted prior to the trial.^22^ ^23^ The intervention was automated and comprised an IVR call^24^ and a pictorial SMS reminder, each sent once a week to the intervention arm participants for 2 years. *The IVR call* said, “Hello, this is your good friend calling you! If you have taken all your medications yesterday please press ‘1’ if no please press ‘2’.” If the patient missed the first call, three additional calls were attempted in the next 24 h. *The SMS reminder* was a neutral pictorial SMS that depicted a ‘lamp’. The SMS reminder was sent to the patient 3 days after the IVR call. Participants could choose a day and time to receive the IVR call and SMS reminder. Participants were trained to receive and respond to the voice calls and access the SMS reminder.

### Data collection

We conducted in-depth interviews with 16 participants in the intervention arm of the trial (5 patients each at the Mysore and Bangalore sites and 6 at the Chennai site). All participants had completed at least 84 weeks since enrollment in the trial. We achieved data saturation around the 16th interview and therefore decided to stop the interviews, as no new information was forthcoming. Purposive sampling included patients from different sites, sexes, occupations, and rural and urban backgrounds.

The first author, who worked with the HIVIND trial and the intervention, but not with routine patient care, conducted the in-depth interviews. All interviews were conducted in a quiet room at each of the study sites and audio-recorded with the participants’ consent. Each interview was conducted in the local language, that is, Kannada or Tamil, and took approximately 20 min. We used an interview guide that was developed and pilot tested to explore participant perceptions of the intervention under specific domains, that is, helpfulness of the intervention, frequency, ease of use, disclosure of HIV status and stigma thereof, preference between the IVR call and the SMS reminder.

### Data analysis

A native local language expert, also proficient in English, transcribed and translated the interviews into English. The first author subsequently spot checked the transcriptions to ensure consistency with the recordings. The thematic ‘Framework Approach’^25^ was used in the analysis. For this, the first author familiarised herself with the transcripts and identified codes. The codes from different transcripts were compared for similarity and grouped under a single category. Codes and categories formed the preliminary framework for the analysis (box 1). Discussions between the authors enabled the reorganisation of codes to match the framework.

Box 1 **Thematic framework used in the study**

- Helpfulness interactive voice response (IVR)
- Helpfulness short messaging service (SMS)
- Perception of importance of the intervention
- Frequency of the intervention
- Functioning of the intervention
- Privacy
- Intrusion
- Stigma
- Stigma from intervention
- Ways to avoid stigma
- Discontinuation of the intervention
- Voice: Male/female preferences
- Technical knowledge
- Preference between IVR and SMS
- Responding to the intervention
- Reasons for non-response
- Reasons for error in response
- Barriers to adherence
- Communication
- Two-way communication
- Type of message
- Suggestions for deployment

The codes were then applied to the transcripts, that is, the transcripts were indexed and the data charted under the relevant codes in the framework. Additional codes were generated as the analysis progressed. Subsequently, the data were summarised and mapped to identify connections between summaries and to derive subthemes. The subthemes were collapsed under broader themes describing the data (table 1).

**Table 1:** A brief description of the framework used in data analysis

Thematic framework components and quotes	Codes	Summary	Categories	Subthemes	Theme*
*Helpfulness of IVR*	Forgetfulness due to work—reminders help	Phone calls minimised forgetfulness and enabled development of a medication routine, improving adherence. Concern from healthcare provider perceived	Improves adherence	*Different perceptions regarding the helpfulness as a reminder*	1
We will be busy with our work, when we get busy, I feel that the reminder is very important for me take the tablets					
When I get a call at 8:00, I feel happy that a call has come from hospital. I feel people from hospital have called	Perceived concern of the healthcare provider along with. Feels happy with the calls		Healthcare providers concern	*Different perceptions regarding the helpfulness as a reminder*	1
*Helpfulness of SMS*	SMS not liked, the inconspicuous alert tone makes the patient miss the SMS	A general dislike for SMS. The SMS is missed on arrival because of its inconspicuous ring tone. Unintended disclosure of HIV status perceived	SMS disliked	*Different perceptions regarding the helpfulness as a reminder*	1
I never liked SMS, I didn't like it at all. I would be busy during the day. I would never hear its sound at all…I never get to know when SMS comes…					
I delete it on the spot but by chance if someone sees it, “LM arogyam”†, they will question me	Perceive the disclosure of HIV status with the SMS		Fear of disclosure of HIV status	*Preventing unintended disclosure of HIV status*	3
*Perception of importance of the intervention*	Intervention does not matter, patient adherent	The necessity of calls is not seen as patients claim to be adherent without the calls. External cues to support adherence used	Perception of being adherent	*Different perceptions regarding the helpfulness as a reminder*	1
Whether I get a phone call or SMS, it doesn't matter, I have taken tablets regularly. That is more important right?					
By 9.00 sharp, after having my breakfast and while taking the cash for my expenses…I will also take the medicines	Patient takes medicines daily after breakfast		External cues for adherence used	*Different perceptions regarding the helpfulness as a reminder*	1
*Preference between IVR and SMS*	Fear of disclosure of HIV status from the phone call	A preference for phone calls in comparison to SMSs. Phone calls thought to have the potential for disclosure of HIV status	Fear of disclosure of HIV status	*Preventing unintended disclosure of HIV status*	3
Yes, she has read the message and asked who is the ‘arogyam’, no one knows, but if you are calling, whoever is attending the call will come to know about the problem					
The call is sufficient, SMS is not necessary…in the phone call they speak, at least to respect (them) we take the call…If we take the call we have to respond…We do not have to respond to the SMS…	Calls are considered sufficient as they are interactive, SMS considered passive		Interaction in the call preferred to passivity of the SMS	*Engagement: IVR* vs *SMS*	2

*Themes: 1. Perceptions of varying usefulness of the intervention, 2. preference for calls over messages and 3. perceived risk of unintentional disclosure of HIV status.

†The name under which the SMS is delivered, ‘arogyam’ means health.

IVR, interactive voice response; SMS, short messaging service; LM, prefix to the alpha numeric sender identifier ‘arogyam’ L- code for the service provider, M- code for the service area. This prefix follows the regulations for SMS sender identification issued by the Telecom Regulatory Authority of India on 10^th^ December 2008, http://www.trai.gov.in/WriteReadData/Direction/Document/direction10dec08.pdf.

### Ethics statement

Prior to enrolment, patients gave their written consent to participate. The consent was also read to all participants in the local language to ensure that they understood the purpose of the study and the study procedures irrespective of their level of literacy.

## Findings

Participant characteristics: Sixteen participants from the intervention arm of the HIVIND trial participated in this study. They were aged 25–56 years (median age: 38 years). Of these, seven were women and nine were men. Of the women, three had part-time employment outside their home. All the men were employed. Six participants had a rural background.

Themes: Three themes emerged from the interviews. These were (1) varying usefulness of the intervention, (2) preference for calls over messages and (3) perceived risk of unintentional disclosure of HIV status.

### Varying usefulness of the intervention

Some participants found that the mobile phone reminders aided establishment of a routine for taking their medication while others did not, citing currently adequate adherence regardless of the intervention. Despite being automated, the intervention was perceived to provide social support and reflect the concern of the healthcare provider.

#### Different perceptions of helpfulness as a reminder

One participant reported that the intervention served as a reminder throughout the day and week despite its bi-weekly frequency. Others reported that the calls minimised forgetfulness, especially when they were busy with work or were away from home.

We will be busy with our work. When we get busy, I feel that the reminder is very important for me to take the tablets … for my health.

The phone calls provided a cue to take medications and helped some participants develop a routine for taking medicines. One patient reported that the embarrassment of providing a negative response to the call ensured her adherence.

In the beginning, I used to forget. So, initially to set the time, this became a good method. I used to miss the tablets initially. Nowadays, I take the tablets at 10:30 in the morning and 10:30 at night. To get in to this routine, this phone call has helped…

Participants who considered themselves adequately adherent to medication did not find the intervention useful. They were aware of the importance of adherence to medication in order to ensure viral suppression. They also reported concern for their own health and a desire to be healthy.

No, (it is not) only because of phone call (that) I remembered to take tablets. It is also because I want to be healthy that I (take) tablets regularly.

Participants suggested sending reminders only to poorly adherent patients that the healthcare provider identified at monthly visits when pill counts were done. They also suggested that the frequency of the reminders could be based on the individuals’ need.

For those irregular with medications you should make the calls every day, those who are regular weekly once is enough.

Reminders were considered necessary for those residing in rural areas, those requiring social support, those preoccupied with work or those with an illness that affected memory.

#### Perceived support: social support and concern of the healthcare provider

The intervention made the participants feel the concern and support from the healthcare provider as a result of the phone calls.

I feel happy that hospital people care for my health, when I receive the call.

The calls were perceived as being from a friend and not from a machine, despite all participants being aware that the calls were automated.

When I get these computerized phone calls asking me how my health is?? I feel contented … Even if I am ready to pay a hundred thousand rupees, I don't think I will get a privilege like this…

### Preference for calls over messages

Despite automating both the IVR call and the SMS reminder, the IVR call was preferred and considered more useful when compared with the SMS reminder. Further, participants rarely viewed the SMS reminder. Unfamiliarity with the concept of SMS combined with its passivity and the inconspicuous alert tone led patients to ignore the SMS reminder.

#### Engagement: IVR versus SMS

The phone call attracted attention and was unlikely to be missed because of the ‘*noise*’ it made while the SMS was easily missed as it made a softer ‘noise’. Participants appreciated the interactivity and technological simplicity of the automated call in comparison to the SMS.

The call is sufficient, (the) SMS is not necessary. If you compare … in the phone call they speak. At least to respect what they speak we pick the call, at least once … We do not have to respond to the SMSs, the SMS is not necessary.

Participants reported a desire to speak to the person calling them. They also wished to discuss their health problems and share their feelings, their disease progression, advice regarding medication and its side effects, nutrition, interactive behaviour and any recent advances in HIV treatment and cure with the caller if given an opportunity

The problem with this phone call is that you cannot speak anything in to it. Only the person on the other side (speaks) …We cannot clarify anything … If that option were there, it would be useful … It would make me feel relieved…

I am a patient, I could have asked detailed information about that about taking tablets, about eating food, how to mingle with everyone, how to work … it may help.

Only one participant reported that two-way communication would be unacceptable, as the person calling would know his disease status.

(A) computerised message is (the) best way. Why should my information be known to the person who is calling?

#### Unfamiliarity with using SMS in the setting

Many participants reported difficulties in accessing the SMS despite being taught. One participant reported that neither he nor his standard 10th educated spouse knew how to view or send an SMS.

Phone call is simple. The SMS is too complicated… I don't understand SMS well…

### Perceived risk of unintentional disclosure from the intervention

Participants found it important to prevent the disclosure of their HIV status, and made efforts to conceal the source of the intervention (ie, the HIV clinic) from others.

#### Preventing unintended disclosure of HIV status

Fearful of others attending the IVR call, participants rarely left the phone unattended on the day of the call. Personalising the time and day of the call enabled participants to choose timings that afforded some privacy and security while answering the call.

No one has picked up the call. Wednesday (I receive) the phone call and (on) Sunday the SMS. On these two days I will never leave the phone anywhere. Even if there is a problem, the phone will be kept with me.

When friends and family enquired about the calls, some participants made attempts to conceal the source of the calls (ie, the HIV clinic).

(When) people around me wanted to know where the call comes from, I tell them that this call is from the Aircel/mobile company and try to escape (the) situation.

Participants considered that frequent calls might increase the risk of unintentional disclosure of their HIV status as the likelihood of someone else receiving the call while the phone is unattended cumulates with the number of calls received.

If I get the call repeatedly, I am scared others will receive the call and will get to know about (my) illness.

Participants reported that they kept the phone to ‘their ears only’ when in the presence of others. One participant reported going out of the house to attend the call, while another personalised the call to receive it after the children had left the house.

Participants deleted the SMS reminders from their phone inbox in an attempt to prevent others from viewing them. One participant feared that if any PLHIV receiving the same SMS reminder viewed his SMSs, his HIV status would be unintentionally disclosed, as the other patient would recognise the SMS.

Although intervention was not considered intrusive, participants either silenced or switched off the phone to prevent the calls from attracting attention in public places.

## Discussion

The results of our study indicate that participants had varied views of the intervention's helpfulness. Some participants perceived the concern of the healthcare provider along with social and emotional support. The IVR call was preferred over the SMS reminder, and participants felt they needed a more engaging call. Participants feared HIV-related stigma, and therefore took steps to reduce the risk of unintended disclosure of their HIV status, via the intervention.

**Figure 3:**
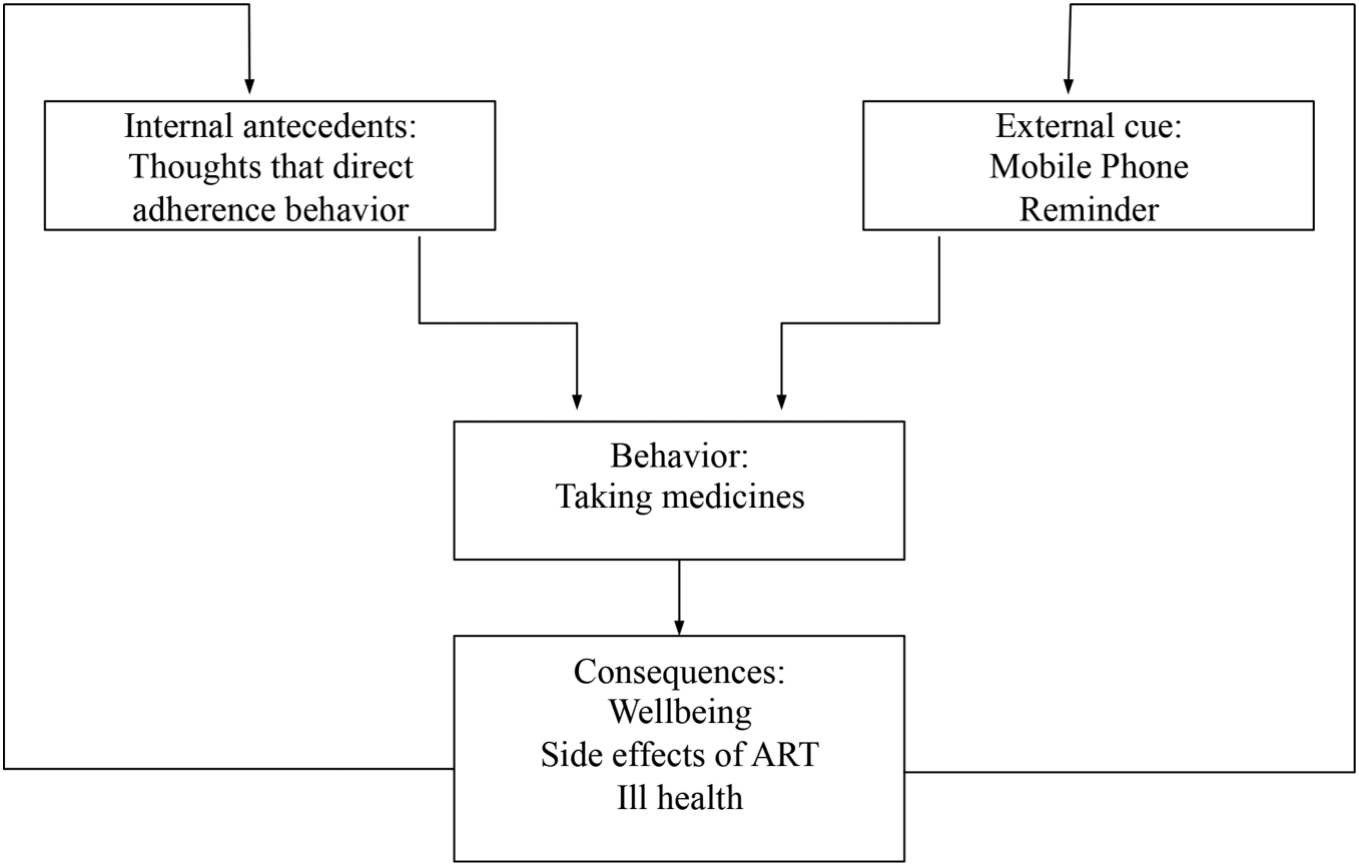
The mHealth antiretroviral treatment (ART) support intervention in the context of the Behavior Learning Theory.

mHealth interventions are designed to improve adherence by targeting forgetfulness, a common barrier to treatment adherence.^26^ Hence, our intervention may be placed within the context of the behaviour learning theory (BLT) framework (figure 3).^16^ The participants in our study desired to stay healthy. This need, an internal antecedent in the BLT framework, probably encouraged them to take medicines. Further, the reminders provided via mobile phones served as an external antecedent. However, studies have shown that external reminders used in isolation may not be able to improve adherence.^27^ ^28^ Effective adherence support interventions need family or peer support, counselling and daily treatment support reflecting their complexity.^28^

Participants also reported feeling the ‘concern’ of their healthcare providers towards their health, more so with the voice calls than with the SMSs. Reports of mHealth interventions providing support and making the recipient feel valued were also observed in Kenya.^5^ ^9^ ^29^ These interventions, however, involved a component of personal interaction via the phone unlike our automated intervention. A study from British Columbia that used text messages in a tuberculosis clinic reported that participants felt supported and cared for by their healthcare provider.^30^ Another study from Peru-related mobile phone reminders to an ‘angel’ and a ‘friend’ giving the intervention an anthropomorphic character.^31^ Similar reports are available from an mHealth smoking cessation trial in the UK.^32^

Despite feeling the concern of the healthcare provider, the participants expressed the need for two-way communication. Engaging beneficiaries in communication improves the efficacy of mHealth interventions.^33^ Two-way communication involving text messages was found to open communication channels and address unmet needs of PLHIV in the Cameroon Mobile Phone SMS (CAMPS) trial. Expressing gratitude, requesting counselling, financial support and advice regarding medication side effects—reasons for using two-way communication in the CAMPS trial—were also mirrored in our study.^34^ Although the content of communication was identified during the design phase in three mHealth trials from sub-Saharan Africa (SSA), none explored the need for interaction.^9^ ^10^ ^13^ Further, though the WelTel and CAMPS trials provided an opportunity for personal interaction with the healthcare provider, only the WelTel trial was successful.^9^ ^10^ The other Kenyan trial was successful even though the intervention was completely automated.^10^ The lack of interaction is therefore unlikely to be a major reason for the negative trial results in our study.

Considering the prevalent literacy levels in our setting, we chose to incorporate a neutral picture SMS in the intervention. Elsewhere, text messages were preferred to other forms of communication as they were considered to provide greater confidentiality.^11^ ^35^ Studies from SSA report high acceptability of text messaging for adherence support, generating awareness regarding HIV infection and communicating laboratory results in HIV-infected individuals.^6^ ^9^ ^13^ None of these studies compared the acceptability of text messages to voice calls. However, another study from South Africa reported nearly equal preference for voice calls and text messages as a mode of communication by a healthcare provider.^36^ A study involving PMTCT indicated that text messages were preferred for brief communications and voice calls for longer, more interactive conversations.^5^ The lower English language literacy in our context in comparison to some of these study contexts could have resulted in the preference for voice calls over text messages. It would be of value to identify the preferred mode of communication and incorporate it into the intervention design. Verbal communication has the potential to overcome the literacy barrier for text messaging, and could be used in low-middle income contexts such as ours. The combined effect of education and text messaging in improving adherence, and the literacy barrier to the use of text messages, reflect the need for alternatives such as voice calls and picture messages for adherence support.^37^

The intervention in our study was designed subsequent to a survey that used a semistructured interview schedule. One in three participants in the survey indicated a strong preference for voice calls. However, the survey did not explore the preferred content of communication or the extent of its interactiveness. Qualitative exploration of participant perceptions while piloting the HIVIND trial intervention could have helped identify and address issues before the trial commenced. Also, those who participated in our study were exposed to the intervention for approximately 2 years. First-hand experience with the intervention probably enabled participants to identify more ‘negatives’ regarding the SMS reminder than were perceived by potential trial participants.

A fear of stigma from an unintended disclosure of their HIV status from the intervention was felt despite the intervention's neutral content, personalised timing and non-verbal interactive communication. Non-verbal communications within the IVR call probably engendered curiosity in those witnessing the call and a fear of HIV status disclosure in the participants. Stigma has been documented as a significant problem for persons living with HIV in the Indian setting.^38^ This fear-induced participants to keep their phones with them on the days that they received the call. They also preferred the reminders weekly as they could keep the phone with themselves for the day. As in other studies, participants in our study also preferred not receiving the calls in public.^36^

Literature on stigma associated with mHealth interventions in HIV infection is largely in the context of text messages. Requests to code content that prevented disclosure of HIV status and minimised stigma were observed in the development phase of the CAMPS intervention.^39^ Stigma as a barrier to mHealth adherence support was identified in the design phase of the WelTel trial.^40^ In our study, the fear of stigma was less pronounced with the SMS reminders than with the IVR calls, probably because the SMS reminders were less popular and went unnoticed more often. As in other studies, participants in our study also resorted to deleting of text messages due to fear of unintended disclosure of HIV status.^6^ The perceived risk of unintended disclosure of HIV status due to others viewing participants’ text messages was also reported from China, SSA and Peru.^13^ ^29^ ^31^ ^36^ ^41^ Researchers should weigh the benefits against the risk of engendering the fear of stigma from mHealth interventions in sensitive health conditions like HIV infection. Educating patients and adequate counselling support could overcome the stigma associated with the intervention.^34^

Considerations for the future design of mHealth interventions: Interventions personalised to the beneficiaries’ need should be developed.^32^ Multicomponent interventions could be designed such that two-way communication with physicians/counsellors, along with information on nutrition, medication side effects and advances in HIV care, is incorporated. Although interactive communication may engage patients for longer durations, it needs to be balanced against the resources needed for intervention scale up.

Sensitivity to the disease and sociocultural contexts, given the possibility of stigma if sensitive information were intercepted, is needed. Targeting those with poor adherence, as suggested by participants in our study, could improve the efficacy of mHealth interventions.^40^ Furthermore, qualitative assessments prior to and during a trial can help develop and contextualise such interventions^32^ These considerations could be extrapolated onto chronic non-communicable disease and tuberculosis.^42^

*Methodological issues:* The study sample was purposive and representative of the participants in the HIVIND trial, enabling the transferability of results. Further, a thick description of the study context is provided in the methods section of this manuscript, enabling the reader to judge the applicability of the results in their setting.

To ensure credibility of the findings, the researchers discussed with each other the interview guide, which was pilot tested and modified suitably to ensure that it accurately assessed participant perceptions. Similarly, a consensus was arrived at regarding the framework, codes, themes and subthemes after discussions with the coauthors. Dependability was supported by describing all procedures undertaken in detail in the methods section of this manuscript.

Reflexivity: The first author's understanding of her position as a physician, a public health person and a researcher living and working in the setting enabled her to effectively contextualise the responses of the participants. The multidisciplinary research team involved in the study contributed to a holistic interpretation of participant perceptions that we explored.

The framework approach is ideal to analyse health system related data as it draws on many different traditions to answer a specific research question. Poised between the inductive and deductive approaches, it encourages the analysis of the respondent's interpretation of issues, in the light of the researchers’ experiences, and supports the contextualisation of research findings.^25^

As the HIVIND trial was ongoing at the time of the interviews, the adherence status of the participants was unknown to the interviewer. Hence, we were unable to relate the experiences of the participants to their adherence within the trial.

## Conclusion

mHealth adherence interventions go beyond their intended role to provide a sense of care and support to the recipient. Although automated interventions are impersonal, they could be the solution for scale up. The utility of conserving sparse healthcare resources by automating interventions should be weighed against the effectiveness of a more engaging intervention involving two-way communication. Multicomponent interventions personalised on the basis of timing, content, duration and adherence levels could be more engaging and acceptable to beneficiaries, and mitigate the risk of stigma. Most of all, mHealth interventions should be developed through extensive behavioural research and tested for effectiveness prior to incorporating them within healthcare systems.
